# Budesonide Nebulization in the Treatment of Neonatal Ventilator Associated Pneumonia

**DOI:** 10.12669/pjms.334.12907

**Published:** 2017

**Authors:** Baoqiang Li, Shuzhen Han, Fuzhen Liu, Lijuan Kang, Chuanwei Xv

**Affiliations:** 1 Baoqiang Li, Department of Pediatrics (II), Binzhou People’s Hospital, Shandong, 256600, China; 2 Shuzhen Han, NICU, Binzhou People’s Hospital, Shandong, 256600, China; 3 Fuzhen Liu, Department of Pediatrics (I), Binzhou People’s Hospital, Shandong, 256600, China; 4 Lijuan Kang, Department of Pediatrics (II), Binzhou People’s Hospital, Shandong, 256600, China; 5 Chuanwei Xv, Department of Pediatrics (II), Binzhou People’s Hospital, Shandong, 256600, China

**Keywords:** Budesonide, Newborns, Ventilator associated pneumonia

## Abstract

**Objective:**

To investigate the clinical effect of budesonide nebulization in the treatment of ventilator associated pneumonia of newborns and its safety.

**Methods:**

Forty-five newborns who had ventilator associated pneumonia and were admitted into the Binzhou People’s Hospital between May 2014 and May 2015 were selected and included as an observation group. Moreover, another forty-five newborns who had ventilator associated pneumonia but did not undergo budesonide treatment in 2014 were randomly selected and included as a control group. Patients in the observation group were given budesonide suspension nebulization in addition to the conventional treatment. The evaluation indicators for therapeutic effect were compared between the two groups. The changes of head circumference, height and weight and death rate were observed by follow up after treatment.

**Results:**

The mechanical ventilation time, time for recovering from chest X-ray scan and hospitalization time of patients in the observation group were shorter than that of the control group, and the difference had statistical significance (P<0.05). The oxygen index of the patients in the observation group was significantly improved compared to that of the control group, and the difference had statistical significance (P<0.05). Patients in the two groups were followed up for six months after discharge. The head circumference, height and weight of the patients in the observation group in the 3^rd^ and 6^th^ month were compared to those of the control group, suggesting no significant differences (P>0.05). The cumulative death rate of the observation group in the 6^th^ month after treatment was significantly lower than that of the control group, and the difference had statistical significance (P<0.05).

**Conclusion:**

Treating ventilator associated pneumonia of newborns with budesonide nebulization can effectively shorten mechanical ventilation time, time for recovering from chest X-ray scan and hospitalization time, improve pulmonary diffusion function and reduce the death rate, without affecting the growth and development of patients in the future.

## INTRODUCTION

Ventilator associated pneumonia is a common complication among patients undergoing mechanical ventilation and one of the main form of hospital-acquired infection.^1^ Previous studies suggested that, the incidence and mortality of ventilator associated pneumonia was 5%~28% and 24% (even 57%) respectively among children who underwent mechanical ventilation,^2^,^3^ and ventilator associated pneumonia is one of the major causes for the extension of length of hospital stay, increased use of antibacterial drugs and expense increase.^4^ The pathogens of ventilator associated pneumonia are multi-drug resistant bacteria such as Pseudomonas aeruginosa, Acinetobacter and methicillin-resistant staphylococcus aureus.^5^ The effect of systemic administration may not be satisfactory as they are usually not sensitive to conventional antibiotics. Inhaled aerosol therapy can directly deliver drugs to lesions through the airway, which is beneficial to improving local drug concentration in lesions and maximize drug efficacy.

Budesonide, a non-halogenated glucocorticoid, can reduce vascular permeability, inhibit secretion of mucus, and relieve edema, spasm and pulmonary ventilation. Budesonide which is inhaled after nebulization can spread to the whole lung and has a high lung deposition rate; budesonide nebulization has lasting effect and is highly effective to local inflammation; hence, it has been applied in the clinical treatment of bronchopneumonia and bronchial asthma.^6^-^8^ But the above method lacks support from clinical evidences concerning ventilator associated pneumonia of newborns. Hence this study used budesonide nebulization to treat ventilator associated pneumonia of newborns as an adjuvant treatment and investigated its clinical effect and prognosis, aiming to provide a reference to clinical medication.

## METHODS

The sample size for this study was calculated according to the following formula:


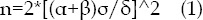


Where δ stands for the required distinction degree, σ stands for overall standard deviation or its estimated value s, and α and β stand for u values which can be searched out in the row of degree of freedom υ∞ in t critical value table. α has bilateral and unilateral values, while β only has unilateral value.

Forty-five newborns who developed ventilator associated pneumonia after ventilator assisted ventilation and were admitted to the Binzhou People’s Hospital between May 2014 and May 2015 were included. There were 24 males and 21 females. Of the 45 cases, there were 25 full-term infants and 20 premature infants. Their age ranged from 30 to 41 weeks (average 38.2±1.51 weeks) and weighed from 1.38 to 4.0 kg (average 2.71±0.84 kg). As to primary diseases, there were 22 cases of neonatal respiratory distress syndrome, 11 cases of neonatal meconium aspiration syndrome, four cases of severe asphyxia, one case of non-cyanotic congenital heart disease and 7 cases of frequent apnea. Forty-five patients were selected from the newborns who had ventilator associated pneumonia but did not undergo budesonide nebulization and were admitted to our hospital in 2014 using random number table and set as a control group. Therefore 25 males and 20 females; there were 23 full-term infants and 23 premature infants. Their age ranged from 31 to 41 weeks (average 37.8±1.74 weeks) and weighed from 1.6 to 3.8 kg (average 2.68±0.71 kg). As the primary diseases, there were 20 cases of neonatal respiratory distress syndrome, 12 cases of neonatal meconium aspiration syndrome, three cases of severe asphyxia, two cases of non-cyanotic congenital heart disease and 8 cases of frequent apnea. The difference of gender, gestational age, birth weight and primary diseases between the two groups had no statistical significance (P>0.05).

### Diagnostic criteria

The diagnostic criteria of ventilator associated pneumonia were new or progressive lung infiltrating shadows by lung imageological examination after no less than 48 hour of mechanical ventilation or within 48 hour after extubation and moreover at least two of the following conditions are satisfied: temperature >38°C or <36°C; count of white blood cells >10×10^9^/L or <4×10^9^/L; there was purulent secretion in the airway. The research protocol had been approved by the ethics committee of the hospital, and the family members of all children have signed informed consent.

Children in the two groups were assisted to breath using MAQUET Serroi which was produced by Germany and whose pointers satisfied the criteria of Practical Neonatology. Children in the observation group were given oxygen-driven budesonide (Astra Zeneca Pty Ltd., Australia, Registration No.: H 20090902) nebulization, 0.5 mg each time, twice each day; oxygen flow was 6~8 L/min, and nebulization lasted for 10 to 15 minutes each time, from the moment of the diagnosis of ventilator associated pneumonia to the 3^rd^ day after ventilator weaning.

### Observation indicators

The mechanical ventilation time, time for recovering from chest X-ray scan, oxygen index before and after treatment and hospitalization time were compared between the two groups. Korean 50mA mobile bedside shoot apparatus (43 KV, 6-10 mas) was used, once every two days; the distance was 60 ~ 90 cm and the exposure time was 0.05 s ~ 0.08 s. Children in the two groups were followed up for six months after being discharged from the hospital. The head circumference, height, weight and cumulative death rate in the 6^th^ month after treatment were compared.

### Statistical analysis

SPSS ver. 20.0 was used for statistical analysis. Measurement data were expressed as mean±standard deviation (SD). The mean was compared between groups using independent sample t test. Enumeration data were processed by Chi-square test. Death rate was analyzed by Kaplan-Meier curve. Difference had statistical significance if P<0.05.

## RESULTS

Airway secretion was bacteriologically cultured on the day diagnosis was made. One hundred and one strains of bacteria were separated from the newborns, including 113 Gram-negative bacteria (84.3%) (22.4% of Pseudomonas aeruginosa, 20.9% of Acinetobacter baumannii, 20.1% of Klebsiella pneumoniae, 15.7% of Escherichia coli and 6.2% of Stenotrophomonas maltophilia). Amikacin was highly sensitive to the separated Gram-negative bacteria, and all the bacteria were sensitive to polymyxin.

### Comparison of clinical observation indicators between the two groups

The mechanical ventilation time, time for recovering from chest X-ray examination and hospitalization time of the observation group were shorter than those of the control group, and the difference had statistical significance (P<0.05). The oxygen index of the two groups suggested no remarkable difference before ventilation (P>0.05). After treatment, the oxygen index of both groups improved, especially the observation group, and the difference had statistical significance (P<0.05) (Table-I).

**Table-I:** Comparison of clinical observation indicators between the two groups.

*Group*		*Observation Group*	*Control Group*	*t*	*P*
Mechanical ventilation time (d)		4.81±2.24	6.18±2.11	2.503	<0.05
Time for recovering from chest X-ray examination (d)		6.96±2.21	8.22±2.13	2.204	<0.05
Hospitalization time (d)		11.41±2.57	14.25±3.33	3.287	<0.05
Oxygen index	Before Ventilation	127.3±15.58	126.4±17.27	0.247	>0.05
	Before Extubation	292.24±31.97	276.48±17.36	2.336	<0.05

### The comparison of follow-up condition between the two groups

Children in the two groups were followed up for half a year after being cured and discharged from the hospital. The follow-up results demonstrated that, there was no difference in the head circumference, height and weight between the two groups in the 3^rd^ and 6^th^ month (Table-II).

**Table-II:** Comparison of follow-up results of growth and development between the two groups.

	*Group*	*Observation Group*	*Control Group*	*t*	*P*
3^rd^ month	Head circumference (cm)	41.08±0.97	41.04±0.95	0.136	>0.05
	Height (cm)	62.17±1.86	62.09±1.74	0.247	>0.05
	Weight (kg)	6.75±0.62	6.81±0.74	0.472	>0.05
6^th^ month	Head circumference (cm)	56.32±1.22	55.83±1.48	0.756	>0.05
	Height (cm)	69.42±2.13	68.16±1.41	0.605	>0.05
	Weight (kg)	8.25±0.60	8.24±0.57	0.532	>0.05

***Comparison of death rate between the two groups***

**Fig. 1:**
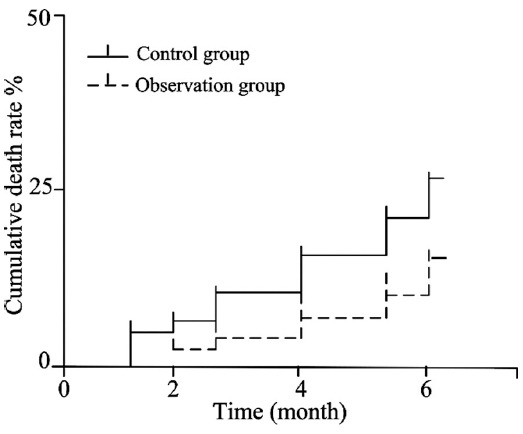
The cumulative death rates of the two groups in the 6^th^ month after treatment.

The death rate of the observation group was 13.3% in the 6^th^ month after treatment, which was significantly lower than that of the control group (26.7%), and the difference had statistical significance (P<0.05) (Fig. 1).

## DISCUSSION

Ventilator-associated pneumonia involves multiple high-risk factors, and its complex pathogenesis is caused by external and internal environment. An single-factor analysis^10^ suggested that premature infants, low body weight, mechanical ventilation time, primary pulmonary diseases, reintubation and with or without use of large-dose gamma globulin was associated to ventilator-associated pneumonia. Therefore how to effectively prevent and treat neonatal ventilator associated pneumonia has been one of the serious issues in clinical practice.^11^ Glucocorticoids have been applied in clinical treatment as it has strong anti-inflammatory effect and can obviously inhibit inflammation induced by different causes and different stages of immunopathogenesis.^12^,^13^ Currently, the effectiveness of budesonide in neonatal treatment has been frequently reported in China and abroad. Mokra et al. found that,^14^ glucocorticoids can effectively improve the lung function of children with meconium aspiration syndrome, relieve inflammation, and partially avoid the inactivation of lung alveolar surfactant. Luo HJ et al. found budesonide can improve pulmonary ventilation and diffuse function in the treatment of neonatal aspiration pneumonia.^15^ For premature infants,^16^,^17^ budesonide nebulization can relieve mechanical ventilation induced airway inflammation and prevent the incidence of bronchopulmonary dysplasia.

The anti-inflammatory effect produced by budesonide nebulization is similar to systemic use of hormone drugs. As the drug can directly produce effect on local sites, the dose needed is smaller, and the coordination of children is also not necessary. Therefore, the longer the duration of nebulization, the higher the drug concentration in the respiratory tract, which makes the treatment effect better than systemic medication. The research results demonstrates that, the mechanical ventilation time, time for recovering from chest X-ray examination and hospitalization of the observation group were shorter than those of the control group (P<0.05), which was consistent with the research results of Cao GK et al.^18^; the oxygen index of the observation group had an obvious increase compared to the control group (P<0.05), suggesting budesonide nebulization could effectively relieve pulmonary inflammatory reaction of neonatal ventilator associated pneumonia, shorten mechanical ventilation time and hospitalization time, and improve pulmonary diffusion function.

The influence of hormones on the hypothalamic-pituitary-adrenal axis is always concerned by both doctors and patients. This study followed up all cured children for half a year. The investigation results demonstrated that, the body development of children in the two groups had no remarkable difference in the 3rd and 6th month (P>0.05), suggesting small-dose budesonide nebulization had a high therapeutic index and high safety and had no adverse effect on the growth and development of children in short time. The cumulative death rate of the observation group was significantly lower than that of the control group in the 6^th^ month after treatment, and the difference had statistical significance. The result might be associated with the effective control of levels of inflammatory cytokine and pulmonary infection; however, the specific mechanism remains to be further investigated.

### Limitations of the Study

The sample size is small. Therefore, multi-center clinical randomized control studies are expected to draw conclusions by means of evidence-based medicine or meta-analysis to guide clinical works.

## CONCLUSION

In conclusion, budesonide inhalation treatment is effective in treating neonatal ventilator associated pneumonia. It is worth promotion as it can accelerate the recovery of children and has high safety and reliability.

### Authors’ Contribution

**BQL& SZH:** Study design, data collection and analysis.

**FZL & LJK:** Manuscript preparation, drafting and revising.

**BQL &LJK:** Review and final approval of manuscript.
